# DeltaProt: a software toolbox for comparative genomics

**DOI:** 10.1186/1471-2105-11-573

**Published:** 2010-11-23

**Authors:** Steinar Thorvaldsen, Tor Flå, Nils P Willassen

**Affiliations:** 1 Department of Mathematics and Statistics, University of Tromsø, 9037 Tromsø, Norway; 2 Norwegian Structural Biology Centre, University of Tromsø, 9037 Tromsø, Norway

## Abstract

**Background:**

Statistical bioinformatics is the study of biological data sets obtained by new micro-technologies by means of proper statistical methods. For a better understanding of environmental adaptations of proteins, orthologous sequences from different habitats may be explored and compared. The main goal of the DeltaProt Toolbox is to provide users with important functionality that is needed for comparative screening and studies of extremophile proteins and protein classes. Visualization of the data sets is also the focus of this article, since visualizations can play a key role in making the various relationships transparent. This application paper is intended to inform the reader of the existence, functionality, and applicability of the toolbox.

**Results:**

We present the DeltaProt Toolbox, a software toolbox that may be useful in importing, analyzing and visualizing data from multiple alignments of proteins. The toolbox has been written in MATLAB™ to provide an easy and user-friendly platform, including a graphical user interface, while ensuring good numerical performance. Problems in genome biology may be easily stated thanks to a compact input format. The toolbox also offers the possibility of utilizing structural information from the SABLE or other structure predictors. Different sequence plots can then be viewed and compared in order to find their similarities and differences. Detailed statistics are also calculated during the procedure.

**Conclusions:**

The DeltaProt package is open source and freely available for academic, non-commercial use. The latest version of DeltaProt can be obtained from http://services.cbu.uib.no/software/deltaprot/. The website also contains documentation, and the toolbox comes with real data sets that are intended for training in applying the models to carry out bioinformatical and statistical analyses of protein sequences.

Equipped with the new algorithms proposed here, DeltaProt serves as an auxiliary analysis tool capable of visualizing and comparing orthologus protein sequences. The framework of the algorithms also enables easy incorporation of extra information on structure, if such data is available.

## Background

Does life existing in extreme environments favour particular amino acids in its proteins, and can this preference be interpreted in terms of the physicochemical properties of these amino acids? Comparative bioinformatics [1] is an emerging field in which the information produced from sequence alignments is analyzed to find out more about the essential properties of proteins in diverse living systems. The success of genome technologies depends heavily on the correct statistical analyses of genomic data, and the new interdisciplinary area of statistical bioinformatics aims to modify available classical and nonclassical statistical methods and develop new methodologies. To explore and analyze the flow of experimental data from molecular biology requires efficient flexible data structures and statistical tools designed with the particular challenges of high-throughput data in mind. Statistical bioinformatics deals particularly with questions of modelling, handling and interpretation of this type of data, especially for modern molecular and post-genomic biology.

The novelty of analytical methods used in statistical bioinformatics and the special nature of the data generated by genome technologies, require the development of new software tools. There is a need for computationally powerful and open/expandable tools, which at the same time should be easy to use. DeltaProt was designed as a Matlab™ [2] companion with a set of functions and algorithms for modelling multiple alignments of proteins using a range of methods. The approach has been successfully applied in research on extremophilic organisms [3-6], and is of special relevance for membrane proteins since it is very difficult to obtain structural models of this important class. This is done by applying comparative statistical methods to comparisons of extremophile proteins versus orthologous genes from organisms of normal (e.g. mesophilic) habitats. Statistical analyses are best at distinguishing valid conclusions from random noise.

The information stored in protein sequences can be analyzed at different levels. Features of proteins that can be obtained as a single numerical value, such as volume or amino acid composition, can be easily compared across large numbers of genomes using statistical methods. Additionally, multiple variants of a gene can be compared between genomes by direct alignment to identify substitution patterns. All genes extracted from two or more genomes provide a further level at which comparisons can be carried out. Regardless of the method used, visual representation of the results becomes essential to display and interpret the findings.

**Figure 1:**
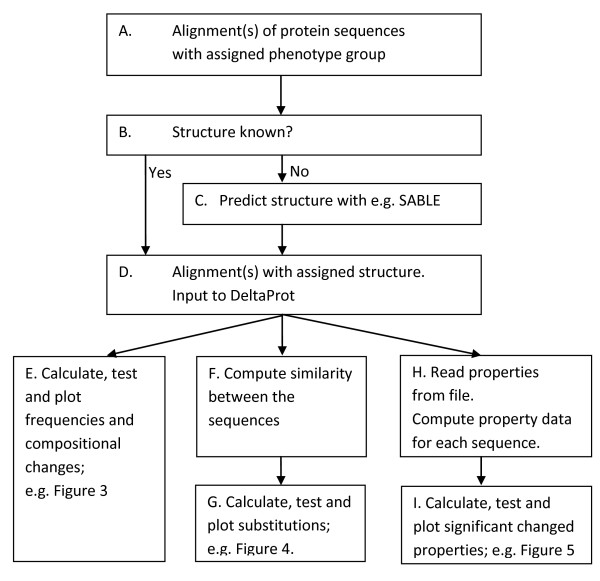
**Flowchart illustrating data use and toolbox functions**. The current version of DeltaProt will handle the process D-I. Analyses may be carried out with various structural constraints imposed on the molecules such as Alpha helixes, Beta sheets, loops, core or surface.

This paper is organized as follows: The implementation section presents the toolbox in more detail by describing the main ideas and capabilities that are integrated into it. This is followed by the results and discussion sections, which summarise the functionality of the software by means of application examples. Figure 1 shows an overview of the workflow. General remarks and future work are also discussed here. The final section of the paper contains a summary of the conclusions.

## Implementation

Here we describe the organization and capabilities of the toolbox, highlighting its key features. This tool provides several methods of comparative analyses, and enables computational scientists to study sequence alignments by rapidly prototyping new algorithms and performing computational experiments. DeltaProt provides access to implementation details and encourages modification and extension of capabilities.

The toolbox is distributed in the standard Matlab language, making it compatible with multiple platforms (Windows, Mac, Linux and Unix). DeltaProt requires at least Matlab™ R14 (2005). Some functions require the Matlab™ Statistics Toolbox. The installation of the package consists of unpacking a compressed file to the desired location. No manual compilation is necessary. The computational requirements are heavily dependent on the size of the data set. Generally, the toolbox only requires high numerical processing capabilities due to a large data set, for example if whole proteomes consisting of more than 1000 alignments, each with 10 sequences, are to be processed.

DeltaProt is supported by a short user manual and a complete webpage with examples, the purpose of which is to facilitate the use of the software for new users. The modular, open-source nature of the software allows users to understand how results are calculated and to add additional functionalities to customized solutions for their own sequence analysis.

### Import of data

**Figure 2:**
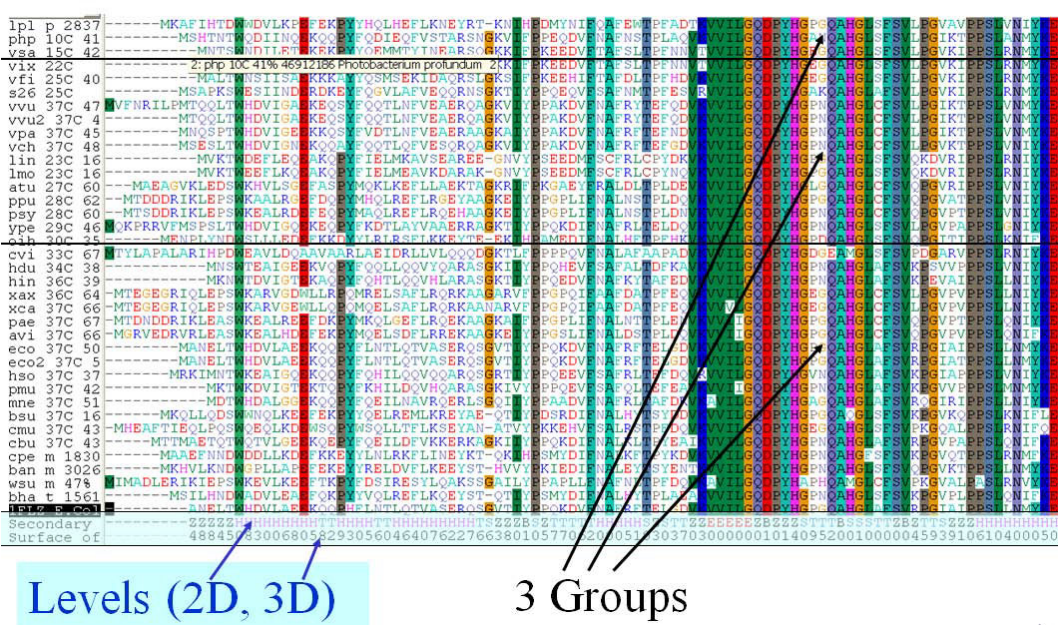
**Input of data**. Input of alignment data from three phenotypic groups in the standard Fasta file format. 2D and 3D contain attached information on secondary structure (2D) and the accessible surface area (3D) of the sites.

The protein sequences must be aligned by using one of the available alignment programs prior to import into and analysis by DeltaProt. DeltaProt may read alignments in standard FASTA file format. Each alignment may also contain extra information on the secondary structure of the amino acids and the accessible surface area (ASA), either obtained from a template structure [7], or from a sequence based prediction program like SABLE [8,9]. Figure 2 shows a window of a typical input file. DeltaProt does not provide any Matlab functions that can help users to create a valid input file containing extra information from the structure predictor. This has to be done manually or by modifying the Python script we have included with the toolbox.

## Results and Discussion

This section outlines the algorithms and performance of the different modules of DeltaProt by considering several illustrative examples of results. Throughout the rest of this paper, we will demonstrate the use of DeltaProt by applying it to a dataset of 65 membrane proteins from six microbial genomes [4]. Users only need to define their sequence data via simple and compact input files. Advanced users can 'tweak' many configuration settings in order to fine-tune for the different data sets.

In DeltaProt we present statistical methods and trend-tests which are useful when the protein sequences in the alignments can be divided into two or more groups based on known phenotypic traits of the host organism, such as preference of optimal growth temperature (mesophile, intermediate, psychrophile) or environmental metagenomic samples. The phenotypic group is assumed to be an ordinal stochastic variable in the statistical model.

The toolbox consists of a set of statistical routines. We consider both the amino acid sequence *compositions *and the *substitution *patterns to determine whether there are underlying trends that explain the observed variation between the phenotypic groups being analyzed. More than 80 different *physicochemical properties *of the amino acid [10,11] may also be applied in order to reduce the sequence alphabet to measurements. Each situation is analyzed by appropriate statistical methods. The differences between the phenotypic groups may also be compared graphically. Amino acid composition, substitution patterns and physiochemical properties along the sequences may all be visualized. The statistical models have a very flexible design, and principles like *pooling *the data to reduce variability by measuring the average instead of the individuals, and *blocking *to reduce the effect of some varying factor that are identifies but uninteresting, may be applied. In cases where data from several different taxonomic orders are available, this information can be utilized, by a *stratified *blocking approach, where the biological order is included in the model. The stratification of the subjects into disjoint sets increases the power of the test to detect association, because like subjects are compared to like subjects.

The approach assumes statistical *independence *among the sequence samples in each group at each strata, but it may in certain cases, be modified to treat phylogenetically dependent sequences within the groups. When there are multiple dependent samples in a group, we compute the mean values in each group at each stratum and apply the statistical test to the means as representative observations.

Both common and more specialized types of non-parametric statistical models [12] are contained in the toolbox, and the models have found good applications in comparative genomics. In Tables 1 and 2 an overview of the available models are briefly presented.

**Table 1:** Statistical models and tests available in DeltaProt for one protein alignment

Groups	Composition	Substitutions	Properties
2	Two-way Anova	Mantel-Haenszel	Linear regression

> 2	Linear regression	_	Linear regression

Sequence data from several taxonomic orders may be utilized as strata in the statistical analysis.

**Table 2:** Statistical models and tests available in DeltaProt to detect common trends in several protein alignments

Groups	Composition	Substitutions	Properties
2	Cumulative Mann-Kendall Wilcoxon paired^§^	Fisher's exact Chi-square^§^	Cumulative Mann-Kendall Wilcoxon paired ^§^

> 2	Cumulative Mann-Kendall	_	Cumulative Mann-Kendall

^§^Tests that should be used if we may not assume statistical independence within each group because of horizontal gene transfer and recombination events. These tests are applied on mean values of each group.

### Composition

**Figure 3:**
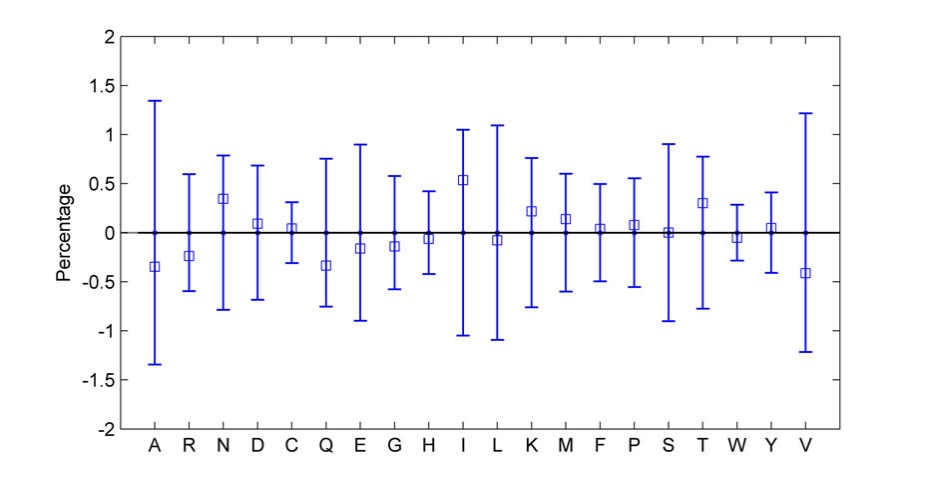
**Composition**. Comparison of the mean amino acid compositional changes calculated on the basis of 65 orthologous membrane proteins [4] observed in the direction from the mesophile to the psychrophile group. Error bars represent the empirical standard deviations. By the cumulative Mann-Kendall trend test the amino acids A, R, N, Q, I, K, T and V have a significant change (p-values < 0.05).

We can examine the change in composition of each amino acid in different phenotypic groups. The amino acids may also be joined in many ways based on common characteristics. Based on charge distribution, one can divide them into four main categories: *negatively charged *(D and E), *positively charged *(K and R), *uncharged polar *(C, S, T, Y, N, Q, W and H) and *hydrophobic *(G, A, V, L, I, F, P and M). The frequency of each amino acid (or other categories) may be analysed by a standard one-way Anova test for data from two groups or linear regression in the case of three or more groups. In addition to temperature, taxonomic order may also be included as the second factor in a stratified model, and two-way Anova (two groups) or regression with dummy variables (with three or more groups) applied. Several proteins may also be analysed to look for common trends. Figure 3 presents a plot of compositional changes observed in our dataset of 65 protein families.

### Substitutions

**Figure 4:**
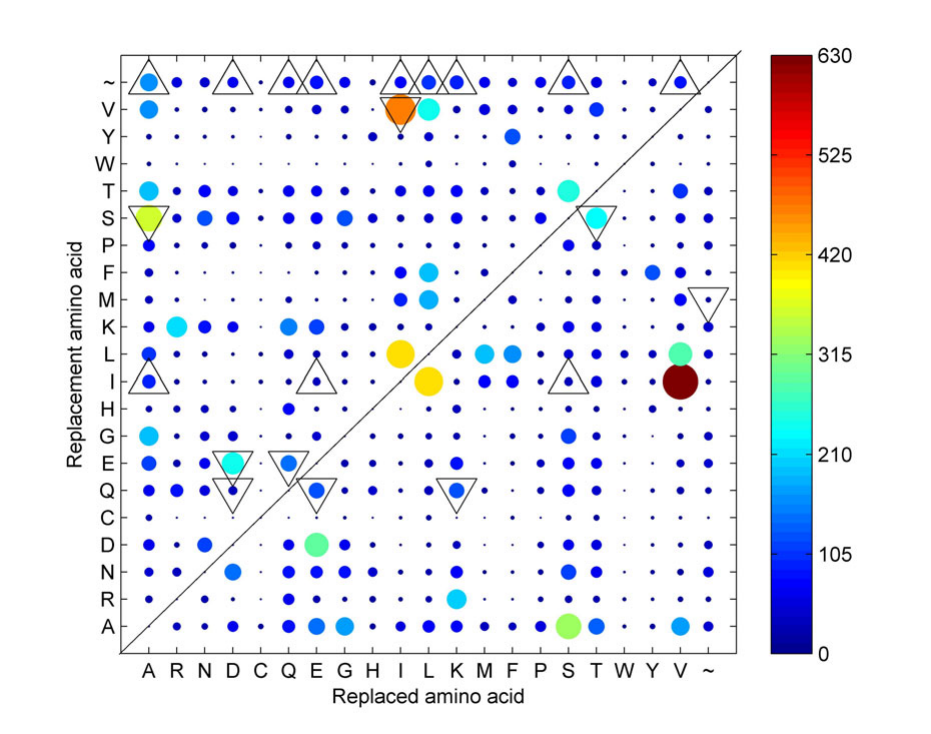
**Substitutions**. Visualization of the number of pairwise substitutions observed in a comparison of 65 orthologous membrane proteins [4] between two groups. The size and colour of each marker indicates the magnitude of the substitution (see colour-bar). A tilde (~) indicates deletion/insertion. Favoured substitutions with p-values < 0.05 in the Replaced (mesophile)→Replacement (psychrophile) direction are marked with upward-pointing triangles, and the non-favoured substitutions with p-values < 0.05 are marked with downward-pointing triangles.

In a comparative study of aligned sequences it is also natural to look for amino acid directional biases in substitutions between two phenotypic groups. We illustrate this type of statistical design with an example. A *Substitution Pair *(SP) is defined as a pair of two amino acids, (*x_M_,y_P_*), where residue *x *of a sequence in mesophilic group *M *is converted to residue *y *in a sequence of the psychrophilic group *P*. For a given pair of amino acids, the "forward" substitution refers to the mesophilic(*M*)→psychrophilic(*P*) direction, and the process may be called cold adaptation. The *SP-matrix *is the count, *n_x,y_*, of all such pairs observed in the alignment by summing over all sites where *x *≠ *y*. This accumulated SP-matrix contains the occurrence of all position-specific pairings of residues. Furthermore, we may calculate the accumulated SP-matrix *between two *groups *M *and *P*, $N_{x,y}^{\left(M,P\right)}$, as well as *within one *group *M*, $N_{x,y}^{\left(M,M\right)}$, where there is no direction. We count by the Jones-method [13], where the sequences with highest identity between the groups are paired and observed amino acid exchanges are tallied in a matrix. The cumulative numbers are found by forming the maximum number of sequence pairs, where each sequence is involved only once to avoid oversampling of the data. Figure 4 presents a plot of all substitutions between two temperature-defined groups.

A natural question to address is whether there are over- or under-representations of amino acid substitutions between the two temperature groups compared to a random model. The number of substitutions of residue *x_M _*→ *y_P _*may be compared relative to several relevant background models (control data) [cf. [4,14]]. For this purpose 2×2 contingency tables may be constructed for each SP of amino acids. In Table 3 the number of forward substitutions (*M→P*) is compared versus the number of background substitutions (*M→ M*). A constant reoccurring problem in statistics is the analysis of 2×2 contingency tables, and there has been a lot of research and debate on this topic [15]. We apply an updated version of Fisher's exact test based on the mid-p-value adjustment for discreteness [16,17] as it is less conservative than standard exact methods, yet usually approximates well the desired error probabilities.

**Table 3:** Substitutions of amino acid x→ y

Substitutions of *x*:	*y*	Not *y*
Adaptation *M→P*:	$N_{x,y}^{\left(M,P\right)}$ **89**	$N_{x,non-y}^{\left(M,P\right)}$ **1754**
Background *M→M*:	$N_{x,y}^{\left(M,M\right)}$ **14**	$N_{x,non-y}^{\left(M,M\right)}$ **514**

A 2×2 table summarizing counts of the SP-matrix for one particular substitution x→y, together with its background. The numerical example in bold is for the substitution A→I of the same data as in Figure 4, with p-value = 0.025 by Fisher's exact test.

The statistical approach above, assumes pairwise independence among the sequence samples (and species) in each temperature group. This is not the case in the presence of underlying phylogenetic processes with horizontal gene transfer and recombination. Hence, we may need to modify the approach to treat each temperature group as one observation. Instead of computing the cumulative SP-matrix, we compute the average countings ${\bar{n}}_{x,y}$ in the representative SP-matrix:

$$\begin{array}{cc} {\bar{n}}_{x,y}^{\left(M,P\right)}=\frac{1}{G_{MP}}\sum n_{x,y}, & x\neq y \end{array}$$

where *G_MP _*is the number of ordered pairs of sequences in the two groups. This use of the group sample mean may also remove some of the random genetic drift in the data.

To be able to detect additional substitution biases, we can also *pool *the substitution data that share the same outcome in the psychrophilic population, e.g. we study the substitutions *x *→ aa, where *x *may be any amino acid and aa is given.

### Regression based on properties

In our case we are basically modeling discrete categorical data. However, the amino acids have many different physicochemical properties, and the amino acid alphabet can be efficiently reduced to univariate measurement data based on the physicochemical properties of each acid, one at a time. The mean property value *Y *is computed for each protein sequence and analyzed by regression, with the phenotypic traits of the host organism as the predictor variable *X*. The phenotypes may be grouped in an ordinal stochastic variable, i.e. preference of optimal growth temperature given as mesophile(*X *= 1), intermediate(*X *= 2), and psychrophile(*X *= 3) [5].

**Figure 5:**
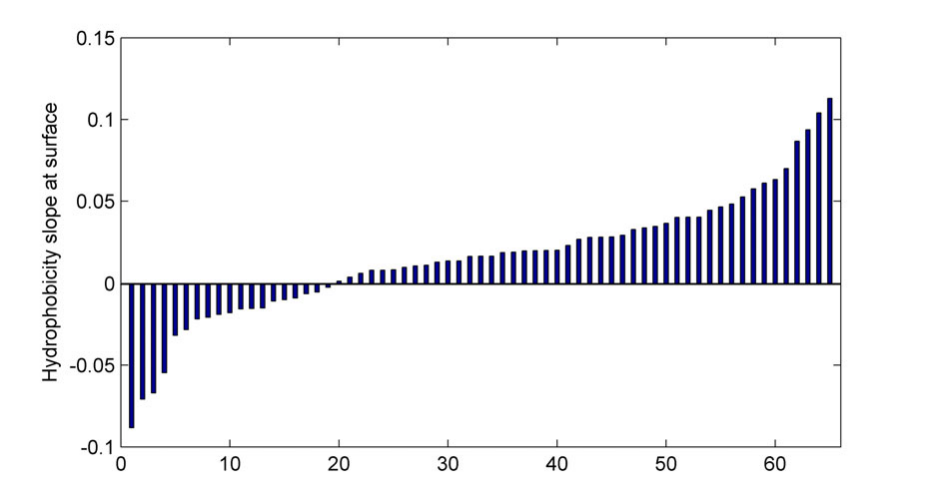
**Slope coefficients**. The ranked distribution of slope coefficients for change of hydrophobicity at the predicted surface [8,9] of 65 membrane proteins [4] as estimated from the regression model. Each of the alignments consists of 6 sequences from 3 phenotypic groups. Computed p-value = 0.00003 from the cumulative Mann-Kendall trend test.

In the case where we have alignments from *several *protein families from a set of genomes and wish to look for common adaptive trends, *Y *can be described by a regression model of the form:

$$Y=\beta_{0}+\beta_{1}\cdot X+e$$

where *e *is the error term. We may include data from several protein families in this model and analyze the final model by applying non-parametric regression based on cumulative Mann-Kendall statistics [5,12]. Figure 5 shows the ranked distribution of estimated slope coefficients *β_1 _*for the data set of 65 protein families.

DeltaProt may also be used to analyze *a single *protein family, by ordinary linear regression. Often there are more sequence data available when we consider just one particular protein. If the data sequences originate from several taxonomic orders of the host organism, this information may be utilized to build a stratified approach. By this method, taxonomic order is included in the regression model as a dummy (or indicator) variable to take into account possible differences due to taxonomy. A linear regression model based on a common trend for all taxonomic orders (parallelism) has the form:

$$Y=\beta_{0}+\beta_{1}\cdot X+\sum_{\text{All }s}\beta_{s}I_{s}+e$$

where *β_0 _*is the intercept term of the baseline taxonomic order, and *β_s _*are the additional strata-specific intercept term for dummy variable *I_s_*.

### False discovery rate

DeltaProt creates an output of summary statistics. These summary statistics include mean, standard deviation and p-values from the comparative tests. When tests of multiple hypotheses are carried out, the significance levels should be adjusted to account for the increased probability of false positives. For multiple test correction, the False Discovery Rate analysis (FDR) of Benjamini and Yekutieli [18] may be applied.

### Limitations

The code for running DeltaProt is entered through a command line interface (CLI) to Matlab, and some key input such as the phenotypic group assignment of the sequences in the alignment(s) must be hard-coded in the source code. Biologists are often unfamiliar with programming computers and interacting with CLIs, and eventually a graphical user interface (GUI), may facilitate the practical use of the software. The approach we use is also suitable for high-throughput sequence analysis through the CLI. Therefore, for large datasets it would be useful to develop a more automatic manner to handle the whole process, from aligning sequences to statistical analysis. We have not ported our application to other similar software platforms like Octave, Scilab or R. With respect to future work on the software, it would also be useful to include analyses of DNA-sequences with physicochemical properties of nucleotides [19,20].

### Similar software

The well known program WebLogo [21] generates a graphical representation of an amino acid or nucleic acid multiple sequence alignment, where each logo consists of stacks of symbols, one stack for each position in the sequence. WebLogo contains no statistical analysis, however Blogo [22] is the same kind of web-based tool that detects and displays statistically significant position-specific sequence bias with reduced background noise. Some other toolboxes have been developed in Matlab for bioinformatic-related analyses. These include MBEToolbox [23,24] and the bioinformatics toolbox developed by Mathworks [2]. MBEToolbox can aid in the exploration, interpretation and visualization of data in molecular biology and evolution. It includes statistical and sequence manipulation functions relevant to molecular evolution, phylogeny inference, and relevant graphic plots. The bioinformatics toolbox from Mathworks offers a wide range of general bioinformatics functions, including molecular evolution.

DeltaProt provides a range of specialised methods which are more relevant to comparative genomics than those included in either of the other projects, and as such it makes a contribution to the bioinformatics analyses that can be performed in the Matlab environment. It may be used to examine if physicochemical differences between proteins may be related to differences in the environment of the source organisms rather than to phylogeny.

## Conclusions

Multiple alignments play a key role in bioinformatics, and provide a very rich set of interesting statistical and data-analysis problems. Addressing these problems will require both development of effective statistical methods and distribution of the implementation of those methods to biologists generating the data. Here we have presented DeltaProt, a toolbox for statistical and visual analyses of alignments from the domain of comparative molecular biology, and we believe that it may provide a good software environment in which to carry out some of this research related to extremophilic organisms. These are organisms that are able to tolerate and thrive in extreme conditions with respect to for example salt, pH, toxic chemicals, radiation, starvation, pressure and temperature. Adaptation to multiple extreme environments (polyextremophiles) makes untangling and isolating the adaptive mechanism(s) to a particular environment even more difficult. Statistical bioinformatics accounts for the inherent variation found in biological data that is generated as part of the bioinformatics investigation, and aims to statistically identify those significant changes in data that answer biological questions. DeltaProt is fully accessible within Matlab and capable of analyzing orthologous protein sequences in seconds. The toolbox is able to handle a large quantity of multiple-aligned sequences in order to look for common adaptive trends at the molecular level. Users can control extra structural attributes, as well as drawing details, to get sufficiently customized output.

## Availability and requirements

*Project name: *DeltaProt, a Matlab toolbox for comparative genomics

*Project homepage: *The toolbox can be downloaded from the following websites, which also offer documentation (installation instructions, manual and tutorial): http://www.math.uit.no/bi/deltaprot/ or http://services.cbu.uib.no/software/deltaprot/

*Operating systems: *Cross-platform (dependent on Matlab availability, tested on Windows and Mac).

*Programming language: *Matlab versions 7.1-7.10 (2010a) is required.

*Other requirements: *Matlab Statistical Toolbox.

*License: *The toolbox can be obtained and used for free for academic purposes, and is under the creative commons license. The conditions of the license can be found on: http://creativecommons.org/licenses/by-nc-sa/3.0/

*Any restrictions to use by non-academics: *Following the previous license.

## Competing interests

The authors declare that they have no competing interests.

## Authors' contributions

ST designed and implemented the software. TF and NPW initiated and supervised the project. ST wrote the manuscript, and all authors have read and approved the final manuscript.
